# Relationship between structure and molecular interactions in monolayers of specially designed aminolipids†

**DOI:** 10.1039/c9na00355j

**Published:** 2019-07-23

**Authors:** Cristina Stefaniu, Christian Wölk, Gerald Brezesinski, Emanuel Schneck

**Affiliations:** Departments of Biomaterials and Biomolecular Systems, Max Planck Institute of Colloids and Interfaces Am Mühlenberg 1 14476 Potsdam Germany stefaniu@mpikg.mpg.de; Institute of Pharmacy, Research Group Biochemical Pharmacy, Martin-Luther-University Wolfgang-Langenbeck-Strasse 4 06120 Halle (Saale) Germany

## Abstract

Artificial cationic lipids are already recognized as highly efficient gene therapy tools. Here, we focus on another potential use of aminolipids, in their electrically-uncharged state, for the formation of covalently cross-linked, one-molecule-thin films at interfaces. Such films are envisioned for future (bio-)materials applications. To this end, Langmuir monolayers of structurally different aminolipids are comprehensively characterized with the help of highly sensitive surface characterization techniques. Pressure-area isotherms, Brewster angle microscopy, grazing-incidence X-ray diffraction and infrared reflection–absorption spectrometry experiments provide a detailed, comparative molecular picture of the formed monolayers. This physico-chemical study highlights the relationship between chemical structures and intermolecular interactions, which can serve as a basis for the rational design of cross-linked thin films with precisely controlled properties.

## Abstract 2

Surface characterization techniques reveal relationships between chemical structures and intermolecular interactions in Langmuir monolayers, serving as a basis for the design of cross-linked one-molecule-thin films with tunable properties.

## Graphical abstract

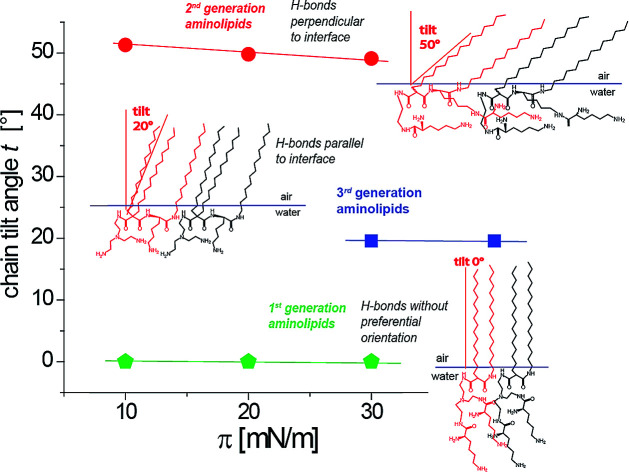

## Introduction

**Fig. 1:**
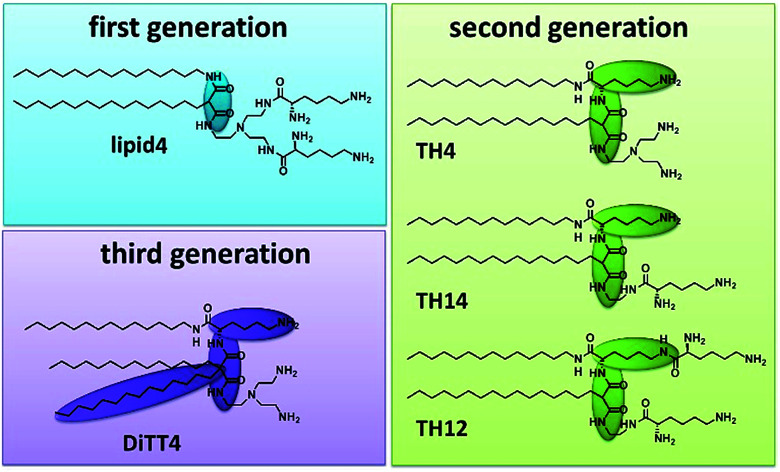
Structures of the investigated aminolipids (lipid4, TH4, TH14, TH12, and DiTT4) belonging to three different “generations” of malonic acid diamides. Structural differences in the backbone are highlighted.

Artificial cationic lipids are becoming increasingly important for medical applications. The major use is in the field of gene therapy, a young field of therapeutic regimes based on the action of nucleic acids as drugs (plasmid DNA, siRNA, miRNA, antisense oligonucleotides, CRISPR-Cas9).^1,2^ The most promising non-viral delivery systems for nucleic acids are made from synthetic cationic lipids and polymers.^3^ After the first synthetic cationic lipid was designed by Felgner *et al.* in 1987 (ref. 4) the range of artificial cationic lipids increased drastically.^5–7^ Furthermore, cationic lipids are useful tools in materials science.^8–13^ Malonic acid diamides are cationic lipids developed in our research groups.^14^ The chronology of their development resulted in three “generations” of malonic acid diamides. The first generation exhibits a backbone composed of malonic acid diamide (Fig. 1) and has led to highly efficient lipids for gene transfer.^15,16^ First structural investigations of selected lipids have shown that their aggregation behavior is characterized by their ability to form hydrogen bonds (H-bonds).^17,18^ Structural investigations guided the design of the second generation of malonic acid diamides,^19^ which are characterized by a complex backbone composed of a malonic acid amide unit and a lysine unit (Fig. 1). This backbone results in a peptide-mimicking structure. Investigations of representatives with the smallest head group revealed a strong H-bond network in the headgroup region.^20^ Some representatives are able to form highly efficient nucleic acid carriers.^21,22^ The third generation is characterized by an expansion of the lipophilic part by introducing a third alkyl chain (Fig. 1). The only representative is DiTT4 which exhibits excellent transfection efficiency alone and in mixtures with phospholipids.^23,24^

The present work addresses the structural details of aminolipid monolayers as well as the underlying self-assembling mechanisms, with the aim to lay the basis future work utilizing the ability of these lipids to form covalently cross-linked nanosheets with tunable properties. Indeed, cross-linked nanosheets of lipids^25,26^ are of great interest due to their use as model membranes for fundamental biophysics studies.^27^ Cross-linking increases the stability of supported lipid bi-(or multi)layers and facilitates their characterization by grazing- incidence X-ray diffraction^28^ or other methods. Moreover, a panoply of other fundamental questions is stimulating the research on two-dimensional (2D) polymers for revealing their mechanical, electrical or magnetic properties, given the fact that such cross-linked nanosheets can have sizes of several square centimeters while still representing one single macromolecule.^29^ More applied directions concern their use as protective layers for electronic devices,^30,31^ ‘anti-fouling’ surfaces which hinder protein adsorption,^32^ surface-assisted self-assembly strategies leading to the growth of nanostructures,^33^ or surfaces with super-wettability.^34^ Lipid monolayers with tailored, cross-linkable headgroups, as investigated here, can thus be considered promising building blocks for the functionalization of gas/liquid and liquid/liquid interfaces leading to defined mechanical properties, in terms of bending-, shear-, and surface-dilational elasticity, as well as transport properties.

For our investigations we have selected malonic acid diamides of the three different generations (Fig. 1). Lipid4, a lipid of the first generation, has low transfection efficiency,^16^ and small-angle X-ray scattering experiments demonstrated that it forms liquid-crystalline lamellar phases at room temperature.^35^ From the second generation three different lipids were selected (TH4, TH14, and TH12) which exhibit the same alkylation pattern (two alkyl chains of different lengths: a C14 and a C16 one) but differences in the headgroup. The number of primary amines ranges from 3 (TH4 and TH14) to 4 (TH12). TH12 has three lysine units in the structure whereby the peptide-mimicking character is increased. TH4 was intensively tested regarding its transfection efficiency, which is very low^22,36^ but structural investigations by cryo-transmission electron microscopy showed that the lipid is able to form ribbon and sheet structures which are interesting for material science.^37^ A special lipid is DiTT4, the lipid of the third generation. The ability to transfect cells without co-lipids and the special aggregation feature which is characterized by a pH-dependent switch from lamellar to micellar arrangements makes it an excellent candidate for biomaterial applications.^24^ The present work focusses on deciphering the correlation between the chemical structure of these aminolipids and their intermolecular interactions which dictate the structural organization of the monolayers and the properties of the cross-linked nanosheets derived thereof in the future. For this purpose, the aminolipids depicted in Fig. 1 were studied in Langmuir monolayers formed at the air/water interface, which are versatile and easy-to-handle two-dimensional model systems. The monolayers were investigated by highly sensitive surface characterization techniques such as pressure-area isotherms, Brewster angle microscopy (BAM), grazing-incidence X-ray diffraction (GIXD) and infrared reflection–absorption spectrometry (IRRAS).

## Results and discussion

**Fig. 2:**
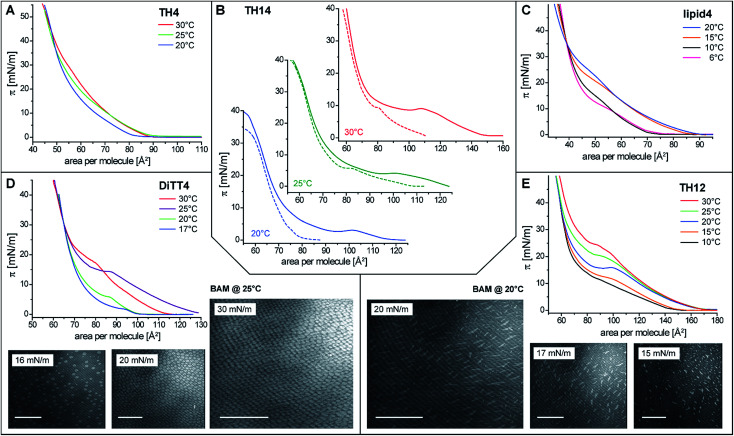
(A–E) Pressure-area isotherms of the studied aminolipids on sodium carbonate–sodium bicarbonate buffers (lipid4 and TH14 at pH 9; DiTT4, TH12, and TH4 at pH 10) at various temperatures. (B) Isotherms of TH14 exhibiting extended pressure plateaus associated with the LE/LC phase transition when the monolayers were spread at 30 °C and the isotherm recorded after longer equilibration time (solid lines) as described in the text in contrast to the standard compression procedure (dashed lines). Panels (D and E) also show Brewster angle microscopy (BAM) images of DiTT4 and TH12, respectively, recorded at various lateral pressures. Scale bar: 100 μm.

In order to gain basic insights into the molecular interactions of the aminolipids and their phase behavior in monolayers, pressure-area isotherms were recorded at various temperatures. Subphases of sodium carbonate–sodium bicarbonate solutions (pH 9 and 10) were used to investigate the monolayer properties under conditions of reduced protonation degree of the free amino groups. These are the optimal experimental conditions for future cross-linking reactions. All isotherms, with the exception of that of TH4 (Fig. 2A), display an inflection point defining the transition pressure *π*_t_ and a plateau region indicating a first-order phase transition from the liquid-expanded (LE) to the liquid-condensed (LC) phase. Coexistence of LE and LC phases in the plateau is clearly seen in the Brewster angle microscopy (BAM) images obtained for DiTT4 and TH12. The nucleation in the monolayer of DiTT4 leads to bright circular LC phase domains (Fig. 2D) surrounded by the dark LE phase, whereas the LC phase domains of TH12 have a needle-like shape (Fig. 2E). All the other compounds (TH4, TH14 and lipid4) form much smaller circular LC domains which are difficult to visualize.

A non-classical behavior was observed for TH14 which displays a larger plateau region after spreading at 30 °C and longer equilibration prior to compression (Fig. 2B). Interestingly, the transition pressure is almost the same in both experiments: 15 min equilibration and measuring at the same temperature or spreading at 30 °C and recording the isotherm after longer equilibration time (20 min at 30 °C + 30 min for reaching the stable lower temperature, 20 or 25 °C). One possible explanation for such an unusual behavior could be the formation of ordered regions in the LE phase already below the transition pressure due to local density fluctuations and based on long-lived intermolecular H-bonds between the aminated headgroups of TH14. Longer equilibration at higher temperatures (30 °C) leads to the (at least partial) rupture of these H-bonds, so that the molecules form a more homogeneous LE phase. The nucleation for those more disordered TH14 molecules starts at larger molecular areas in the LE phase. Since the packing in the LC phase is the same, the phase transition plateaus are much larger (Fig. 2B). A similar behavior is observed for other aminolipids exhibiting a pronounced shift to larger molecular areas in the LE phase whereas the packing in the LC phase is the same (lipid4 at 15 and 20 °C, DiTT4 at 25 and partially 30 °C, and TH12 at 20, 25 and 30 °C (Fig. 2C–E)). Such behavior indicates that the monolayers are not in thermodynamic equilibrium. Usually, the decompression isotherms can be taken as equilibrium isotherms for the thermodynamic analysis.^38^ However, in the present case, the decompression isotherms do not exhibit a plateau region. The formation of strong interactions in the LC phases leads to densely packed layers which transform directly into the gas phase (sublimation) at close to zero pressures. BAM images support the co-existence of solid islands in gas-analogous surroundings.

**Fig. 3:**
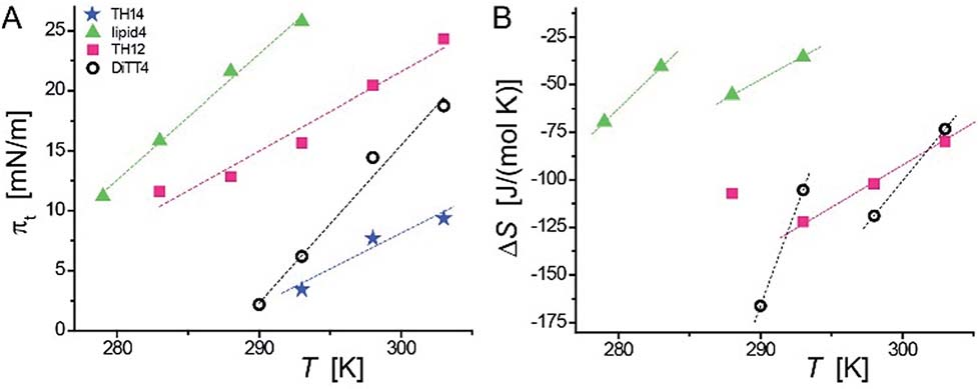
Temperature dependence of the transition pressure (A) and of the entropy change (B) for the LE/LC phase transition measured for aminolipid monolayers on the surface of sodium carbonate–sodium bicarbonate buffers (lipid4 and TH14 at pH 9; DiTT4 and TH12 at pH 10).

Because of the non-equilibrium state of most of the layers, thermodynamic data extracted from the compression Langmuir isotherms using a two-dimensional version of the Clausius–Clapeyron equation,^39^ Δ*H* = (*A*_LC_ − *A*_LE_)*T*d*π*_t_/d*T*, with the molecular area at the beginning (*A*_LE_) and the end (*A*_LC_) of the plateau at the transition pressure (*π*_t_), are only apparent ones and have to be discussed with care. The temperature-dependence of *π*_t_ is presented in Fig. 3A, while the temperature dependence of the entropy change (Δ*S* = Δ*H*/*T*, which is valid at conditions of phase coexistence with Δ*G* = 0) is depicted in Fig. 3B. Negative values of Δ*H* and Δ*S* are obtained, reflecting the exothermic nature of the transition upon compression due to an increase in the monolayer ordering when entering the condensed state.

The Δ*S* values of TH14 will not be discussed because of the opposite temperature dependence preventing the determination of a critical temperature (Δ*S* = 0). However, the apparent Δ*S* values of lipid4, TH12, and DiTT4 can be discussed. They support the scenario of a coexistence of completely disordered and partially ordered regions, likely stabilized by H-bonds, in the LE phase. Due to the non-equilibrium character of the compression isotherms, the absolute Δ*S* values cannot be compared but only relative changes. The obtained data allow to establish a correlation between the chemical structures of the five aminolipids and their thermodynamic properties. Lipid4, belonging to the 1st generation and bearing a quite bulky headgroup, requires the highest compression (*π*_t_ values) to undergo the LE/LC phase transition. This indicates that the LC phase of lipid4 is thermodynamically less favorable than those of the other lipids. This observation is in agreement with the low magnitude of Δ*S* despite the observed smallest molecular areas (Fig. 2C) in the LC phase.

TH12, belonging to the 2nd generation of aminolipids (Fig. 1), is second hardest to compress into its LC phase (Fig. 3A). The headgroup of TH12, which is also bulky, apparently adapts a different orientation at the air/water interface than lipid4, because its molecular area is much larger (see isotherms in Fig. 2). The larger absolute Δ*S* values obtained for TH12 sustain the idea of a higher degree of ordering in the LC phase. DiTT4, the 3rd generation aminolipid, exhibits lower *π*_t_ than lipid4 and TH12 indicating a stabilization of the LC phase. The reason might be additional hydrophobic interactions due to the third alkyl chain, which at the same time leads to a much better match between the space requirements of hydrophilic and hydrophobic moieties.

Interestingly, TH14 reveals the lowest *π*_t_, implying that this compound manifests even more favorable intermolecular interactions, possibly due to the formation of a strong inter-headgroup H-bond network. TH14 bears a smaller headgroup than lipid4 and TH12, and therefore supposedly adopts a different headgroup orientation, which favors the formation of H-bonds and the anomalous temperature-dependence of Δ*S*. The absence of a defined plateau region in the pressure-area isotherms of TH4 indicates a direct phase transition from a gas-analogue (G) to a LC phase. Surprisingly, the molecular areas at low surface pressures are very large for LC phases leading to a model of a condensed phase with many voids. In summary, the above analysis allows for a ranking of the studied aminolipids with regard to the thermodynamic stability of the LC phase: TH4 ≥ TH14 > DiTT4 > TH12 > lipid4.

**Fig. 4:**
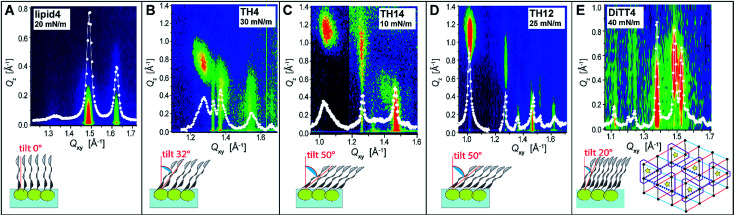
(A–E) (top row) Representative GIXD plots (intensity as a function of the out-of-plane component *Q*_*z*_ and the in-plane component *Q*_*xy*_ of the scattering vector) characterizing the monolayer LC phases of all studied aminolipids. Superimposed as white lines are the *Q*_*z*_-integrated Bragg peak intensities as a function of *Q*_*xy*_. White dots: experimental data; white lines: Lorentzian fits. (bottom row) Schematic illustrations of the chain tilt. Inset bottom right in panel (E): schematic top-view representation of the lattice formed by DiTT4. The positions of chains and headgroups are indicated with black dots and yellow stars, respectively. Red, black, and blue triangles indicate the repeating unit cell of the alkyl chains. The dashed parallelogram indicates the molecular unit cell. Purple line: schematic delimitation of the molecules.

In order to obtain further structural information on the liquid-condensed monolayer phases, GIXD measurements were performed. This method allows determining the lattice parameters of the LC phases at the Angstrom scale (the technical details are described in the ESI†). The good agreement between the molecular areas determined in the isotherms and in the GIXD experiments at high pressures indicates that the monolayers are entirely in the LC phase above the plateau. Fig. 4 depicts the characteristic GIXD contour plots measured for each aminolipid. The diffracted intensity is plotted as a function of the out-of-plane component *Q*_*z*_ and the in-plane component *Q*_*xy*_ of the scattering vector. Superimposed as white lines are the *Q*_*z*_-integrated Bragg peak intensities as a function of *Q*_*xy*_.

The 1st generation lipid (lipid4) exhibits two intense diffraction peaks at different *Q*_*xy*_ but both at *Q*_*z*_ ≈ 0 (Fig. 4A) at all lateral pressures (10 mN m^−1^ ≤ *π* ≤ 30 mN m^−1^), indicating an orthorhombic structure with un-tilted chains (*t* = 0°) and a nearest neighbor (NN) distortion (for detailed GIXD data and lattice parameters see ESI†). The monolayer structure virtually does not change upon compression; however, the correlation length (determined from the *full-width at half-maximum* (fwhm) of the peaks according to the Scherrer equation, see ESI†) is reduced. Namely, the average crystallite size decreases drastically from *A*_cryst_ ≈ 670 nm^2^ to *A*_cryst_ ≈ 70 nm^2^ upon compression from *π* = 20 mN m^−1^ to *π* = 30 mN m^−1^. The presence of the weak and broad Bragg peak at *Q*_*xy*_ = 1.325 Å^−1^ is an indication of a poorly correlated H-bond network established between the headgroups. Sharper and more intense Bragg peaks characterizing a more strongly correlated H-bond network are observed for the aminolipids of the 2nd generation (Fig. 4B–D), especially for TH4. In addition to this peak, all the three compounds belonging to this generation (TH4, TH14 and TH12) exhibit three Bragg peaks associated with the chain lattice, indicating an oblique structure that is essentially insensitive to the lateral pressure. There are, however, distinct differences between the three compounds. While the alkyl chains of TH4 are only moderately tilted (*t* = 32°, see Fig. 4B) with respect to the surface normal, the chains of TH14 and TH12 are strongly tilted (*t* ≈ 50°, see Fig. 4C and D). This behavior is associated with larger molecular in-plane areas for TH14 and TH12 dictated by their bulkier headgroups. The observed insensitivity to the lateral pressure indicates that the headgroup layer is rigidified by the intermolecular H-bonds which ultimately control the molecular packing in the LC phase. The coherence length of the 2D crystallites formed by TH12 is much higher (*A*_cryst_ ≈ 1200 nm^2^) than those of the LC phases of TH4 and TH14 (*A*_cryst_ ≈ 30 nm^2^ and *A*_cryst_ ≈ 130 nm^2^, respectively), in line with the BAM results discussed above.

The LC phase formed by the 3rd generation aminolipid DiTT4 exhibits the most complex structure (Fig. 4E). Similar to the 2nd generation compounds, the DiTT4 monolayer structure is defined by three diffraction peaks above the horizon (*Q*_*z*_ > 0) in the wide-angle region (at high *Q*_*xy*_), characterizing an oblique lattice structure of tilted chains (*t* ≈ 20°, Fig. 4E and Tables S9–S11†). As for all the other aminolipids, no structural changes were observed upon lateral compression to higher surface pressures. Three additional Bragg peaks are present in the mid-angle region (lower *Q*_*xy*_). These peaks, together with the rigid, unchanged monolayer structure, indicate an increased ordering of the headgroups.

A supercell indicating the ordering of entire DiTT4 molecules was identified, induced by the formation of a strong intermolecular H-bond network between the amine-amide headgroups and sustained by the strong hydrophobic interactions of the three alkyl chains, similar to the previously reported monolayer structure of a GPI fragment^40–42^ and other glycolipids.^43^ The supercell (dashed parallelogram in Fig. 4E) is commensurate with the hydrocarbon chain lattice (*b*′ = 2 × *b*_chains_, *c*′ = 3 × *c*_chains_, *γ* = 125.5°) and contains two DiTT4 molecules (*A*_SC_ = 128.2 Å^2^ = 6*A*_xy_, where *A*_xy_ = 21.4 Å^2^ is the in-plane area per chain). The fwhm in *Q*_*z*_-direction of the Bragg rods (ESI†) agrees well with the length of an extended C14 alkyl chain in *all-trans* conformation, confirming that the interfacial layer is a monolayer at all investigated surface pressures.^44–46^ The correlation length of the DiTT4 2D crystallites is the highest one of the series, *A*_cryst_ ≈ 2600 nm^2^ at 30 mN m^−1^ (see ESI† for detailed GIXD tables and lattice parameters).

**Fig. 5:**
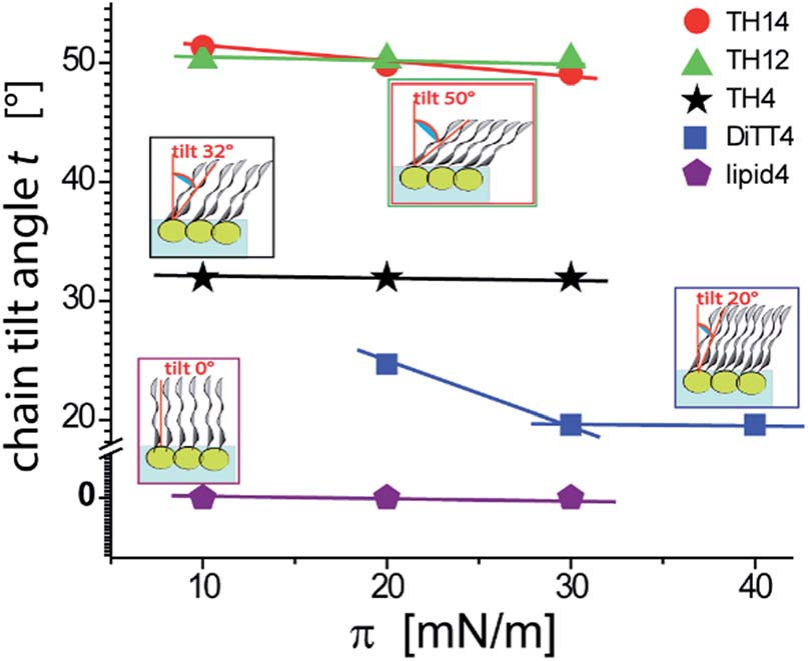
Variation of the chain tilt angle *t* of the aminolipid monolayers with the lateral pressure as obtained by GIXD.

An overview of the chain tilt angle as a function of *π* is given in Fig. 5 for all aminolipids investigated. Lipid4, belonging to the 1st generation, is able to form condensed interfacial monolayers characterized by un-tilted lipid chains. This result demonstrates that the area requirement of the chains is comparable to that of the headgroup, even though this lipid bears the bulkiest headgroup of the series. The headgroup for this purpose has to adopt an upright orientation. With that, the monolayer structure is dictated by the hydrophobic interactions of the alkyl chains. The headgroups are nonetheless able to form H-bonds, as explained above. A low (albeit significant) extent of chain tilting occurs for the 3rd generation aminolipid DiTT4. Interestingly, in this case there is a subtle competition for structural dominance between the hydrophobic interaction of the three chains and the strong H-bond network formed by the headgroup. The aminolipids of the 2nd generation clearly exhibit the strongest headgroup interactions, so that they form LC phases with higher tilt angles. TH4, which bears the same headgroup as DiTT4, forms a LC phase with a higher tilt angle because of the lower area requirement of only two instead of three alkyl chains. The monolayers of TH14 and TH12 are characterized by the largest chain tilt angles (*t* ≈ 50°) due to the strong H-bond network established between the bulky headgroups. The key role played by the spatial distribution of the structural motifs in the headgroup region is highlighted by comparison of the monolayer structures of TH12 and lipid4: despite the fact that the two aminolipids bear almost the same structural motifs in the headgroups (Fig. 1), their different spatial distribution clearly leads to pronounced differences in the packing arrangements, notably regarding the chain tilt angles (*t* ≈ 50° *vs. t* ≈ 0°).

**Fig. 6:**
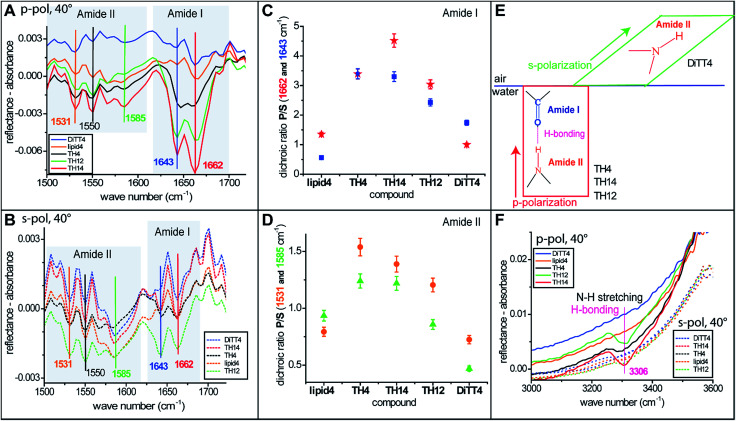
(A and B) IRRA spectra of aminolipid monolayers on the surface of sodium carbonate–sodium bicarbonate buffers (lipid4 and TH14 at pH 9; DiTT4, TH12, and TH4 at pH 10). The graphs show amide I and amide II bands recorded with p- and s-polarized light, respectively, at an incident angle of 40°. (C and D) Dichroic ratio (*I*_p_/*I*_s_) of the amide I and amide II bands, respectively. (E) Schematic representation of the orientation of the H-bonds in a plane perpendicular to the air/water interface – occurring for the 2nd generation of aminolipids (TH4, TH14, TH12) and the preferential orientation of the amine groups in a plane parallel to that of the interface – occurring for the DiTT4. (F) IRRA spectra of aminolipid monolayers showing the amide A bands.

For a better understanding of the headgroup intermolecular interactions and their orientation, IRRAS measurements (the method is described in the ESI†) were performed with monolayers of all the aminolipids. Fig. 6A and B show the amide I and amide II bands recorded with p- and s-polarized light, respectively, at an incident angle of 40°. Two strong amide I bands at 1643 and 1662 cm^−1^ are observed for some of the aminolipids. The major component of this band is the stretching of the C=O bonds.^47,48^ Interestingly, the amide I bands for the lipids of the 2nd generation (TH4, TH14 and TH12) recorded with p-polarized light are more intense than those recorded with s-polarized light (Fig. 6A and B). This is clearly seen in Fig. 6C, which shows the P/S dichroic ratio calculated for the amide I bands, and indicates that the C=O moiety adopts preferentially a perpendicular orientation to the air/water interface (Fig. 6E). The P/S dichroic ratio values close to unity obtained for lipid4 and DiTT4 indicate that in these cases the transition dipole moments of C=O groups are equally distributed in planes parallel and perpendicular to the interface (Fig. 6C). The in-plane N–H bending and the C–N stretching modes manifest in the form of three amide II bands^47^ at 1531, 1550 and 1585 cm^−1^. The associated P/S dichroic ratios (Fig. 6D) are closer to unity than those of the amide I band, indicating only a slight preferential orientation of the C–N–H groups perpendicular to the interface. In contrast, the ratios close to 0.5 obtained with DiTT4 indicate that in this case the C–N–H moiety adopts preferentially parallel orientation to the air/water interface (Fig. 6D and E). Interestingly, only the aminolipids of the 2nd generation exhibit the amide A band at 3306 cm^−1^ (Fig. 6F), which is given by the N–H stretching and a signature of H-bond formation.^47^ The fact that this band was revealed only by p-polarized light is another confirmation that the strong H-bond network formed by the aminolipids of the 2nd generation is preferentially oriented in a plane perpendicular to the air/water interface (Fig. 6E and F).

## Conclusions

**Fig. 7:**
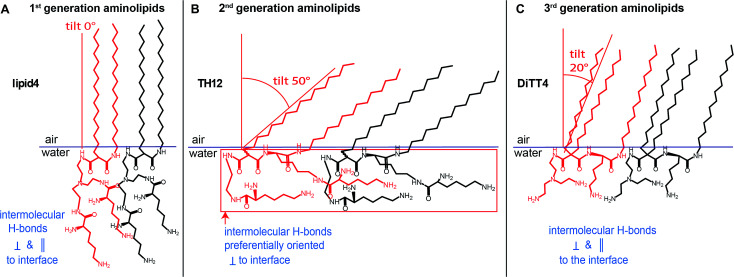
(A–C) Schematic representation of the characteristic features of the LC phases formed by the aminolipids of the three different generations. Highlighted are differences in the tilt angles adopted by the ordered alkyl chains (defining the thickness of the hydrophobic part of the film) and the different orientations of the headgroups with respect to the air/water interface (influencing the thicknesses of the entire films).

In conclusion, this physical-chemical study establishes a correlation between the chemical structure of three generations of aminolipids and their intermolecular interactions manifested in their structural organization in Langmuir monolayers, as summarized in Fig. 7. Such detailed information is essential for their future use in the development of cross-linked stable one-molecule thin films with possible applications in the (bio-)materials fields. The obtained structural data reveal that the thickness of the cross-linked films could be designed and controlled by the proper choice of the aminolipid. Accordingly, the 1st generation aminolipid could form thicker films, while the 2nd and the 3rd generations of aminolipids could form thinner or intermediate films, respectively. Furthermore, the detailed information obtained about the orientation of the headgroups at the air/water interface will shed light on the possible differences occurring in the mechanism of the cross-linking reaction. Thus, this class of substances could offer the possibility of designing one-molecule thin covalently cross-linked films for which the thickness as well as the hydrophilic/hydrophobic thickness ratio can be controlled to an Angstrom level. These characteristics, in turn, determine the layers' permeability for small molecules, as well as their mechanical properties in terms of bending-, shear-, and dilational elasticity.

## Conflicts of interest

There are no conflicts to declare.

## Supplementary Material

NA-001-C9NA00355J-s001
