# Supplementary material for: Relationship between structure and molecular interactions in monolayers of specially designed aminolipids
Source: Nanoscale Adv. 2019 Jul 23;1(9):3529–36. doi: 10.1039/c9na00355j (PMC9418614; doi:10.1039/c9na00355j)
Supplement: NA-001-C9NA00355J-s001 [file NA-001-C9NA00355J-s001.pdf]

Electronic Supplementary Material (ESI) for Nanoscale Advances.
This journal is © The Royal Society of Chemistry 2019

Relationship between structure and molecular interactions in monolayers of specially designed aminolipids

Cristina Stefaniu[a,] Christian Wölk[b], Gerald Brezesinski[a] and Emanuel Schneck[a]*

[a] Departments of Biomaterials and Biomolecular Systems, Max Planck Institute of Colloids and Interfaces, Am Mühlenberg 1, 14476 Potsdam, Germany

[b] Institute of Pharmacy, Research Group Biochemical Pharmacy, Martin-Luther-University, Wolfgang-Langenbeck-Strasse 4, 06120 Halle (Saale), Germany

* Corresponding author: stefaniu@mpikg.mpg.de

EXPERIMENTAL SECTION

Materials

The lipids have been synthetized and the purity proved as previously reported[1-3].

Surface Pressure Measurements

Pressure-area isotherms were recorded on a computer interfaced Langmuir trough with a surface area of 194 cm^2 (Riegler & Kirstein, Potsdam, Germany). The paper plate Wilhelmy method was used to measure the surface tension with an accuracy of ± 0.1 mN/m. Each measurement was repeated at least three times. The Langmuir trough was filled with a sodium carbonate – sodium bicarbonate buffer solutions, pH 9 and/or pH 10. The 1 mM lipid solution in a $CHCl_3$: MeOH mixture 9:1 (vol) was spread and the measurement started 15 min after the evaporation of the solvent. Langmuir Isotherms were measured for each compound at different temperatures of the subphase.

Brewster Angle Microscopy

The morphology of the monolayer was imaged with a Brewster angle microscope, model BAM2plus from NanoFilm Technologie (Göttingen, Germany), equipped with a miniature film balance from NIMA Technology (Coventry, UK), both mounted on an antivibration table. The microscope was equipped with a frequency-doubled Nd:YAG laser (wavelength λ = 532 nm, ≈ 50 mW), a polarizer, an analyzer, and a CCD camera. When p-polarized light is directed onto the pure air/water interface at the Brewster angle (≈ 53.1°), zero reflectivity is observed. The presence of a monolayer causes light to be reflected because of the changed refractive index of

the surface layer, which is then registered by the CCD camera after passing the analyzer. BAM images of 45 × 470 μm^2 were digitally recorded during compression of the monolayer. The lateral resolution was ≈ 2 µm.

Infrared Reflection Absorption Spectroscopy Measurements (IRRAS)[4-6]

The experiments were performed using an IFS 66 FTIR spectrometer equipped with a liquid nitrogen cooled mercury cadmium telluride detector attached to an external air/water reflection unit (XA-511, Bruker). The principle of the method and its application to Langmuir films at the air/water interfaces have been previously described.[6] A small reference trough and the larger sample trough are alternatively moved into the IR beam path by a shuttling mechanism. The measured signal in infrared reflection absorption spectroscopy (IRRAS) represents the ratio of the reflected light from two liquid surfaces:

$$RA = -\log[(\text{sample reflectivity})/(\text{reference reflectivity})] = -\log(R/R_0)$$

in reflectance/absorbance (RA) units. The resolution and scanner speed in all experiments were 8 cm^{-1} and 20 kHz. The incident IR beam is polarized with a KRS-5 wire grid polarizer. Spectra are co-added over 200 scans for s-polarized light and over 400 scans for p-polarized light. The two different light polarizations provide information on molecular orientation with respect to the surface plane of the monolayer. A change in the intensity ratio of p-polarized to s-polarized light (p/s ratio) for a vibrational band indicates a change in the average orientation of the vibration and thus of the molecule.

Grazing incidence X-ray diffraction (GIXD)

The monolayer's lattice structure was investigated by grazing incidence x-ray diffraction (GIXD) measurements performed at the synchrotron beamline P08 at PETRA III of DESY (Hamburg, Germany). The photon energy was 15 keV (wavelength λ = 0.826 Å, P08). Temperature-controlled Langmuir troughs (R&K, Potsdam, Germany), enclosed in a sealed, helium-filled container, were used. The incident beam hits the water surface at a grazing incidence angle θ_i = 0.07° (P08), below the critical angle of total reflection. The diffraction signal is measured with a vertically-oriented position-sensitive (PSD) detector (P08: MYTHEN, PSI, Villigen, Switzerland) scanning the azimuthal angle Δ and, with that, the in-plane component $Q_{xy} = (4\pi/\lambda)\sin(\Delta/2)$ of the scattering vector $\boldsymbol{Q}$. The in-plane divergence of the diffracted beam is restricted to $\delta\Delta$ = 0.09° with a Soller collimator. The out-of-plane component Q_z of the scattering vector is encoded in the vertical position of the PSD channels and covered the range from 0.0 to 1.2 $Å^{-1}$ (P08). The diffraction data consist of Bragg peaks in the 2-dimensional Q_{xy}/Q_z space. Their precise Q_{xy} positions are determined by integration over a defined vertical Q_z-window and subsequent

fitting of the 1-dimensional curves with a Gaussian function. The in-plane lattice repeat distances of the ordered structures then follow from Bragg's law: $d = 2\pi/Q_{xy}$. The tilt of the lattice with respect to the vertical direction is obtained from the Q_z positions of the peaks.[7]

To access the extent of the crystalline order in the monolayer, the in-plane coherence length L_{xy} was approximated from the resolution-corrected full-width at half-maximum,

$$fwhm_{corr}(Q_{xy}) = \sqrt{fwhm^2 - \delta Q_{xy}^2},$$

of the Bragg peaks using the Scherrer equation $L_{xy} \approx 0.9(2\pi)/\text{fwhm}_{corr}(Q_{xy})$. The resolution $\delta Q_{xy} = 0.012$ Å^{-1} follows from the angular divergence $\delta\Delta$ as $\delta Q_{xy} \cong (4\pi/\lambda)\sin(\delta\Delta/2)$. The average crystallite size was roughly estimated as the area of a parallelogram defined by the maximal and minimal coherence length, L_{xy}^{max} and L_{xy}^{min}, respectively having a relative angle of 60° in an approximately hexagonal arrangement:

$$A_{cryst} = L_{xy}^{max} L_{xy}^{min} \sin(60°).$$

The diffracted intensity normal to the interface was integrated over the Q_{xy} window containing the diffraction peak to calculate the corresponding Bragg rod. The thickness of the monolayer is estimated[7] from the fwhm of the Bragg rod as $\approx 0.9(2\pi)/\text{fwhm}(Q_z)$.

Peak indexing for superstructures

We used a previously reported procedure to reconstruct the crystalline superstructures of the headgroup-ordered monolayers[8]. The primitive unit cell vectors describing the oblique lattice of the hydrocarbon chains were defined according to the parameters in the GIXD tables below. The vectors ***a*** and ***b*** (see Fig. 2D) are associated with the reciprocal space vectors ***a***** and ***b*****, as in the book by Kittel[9], Chap. 2. The reciprocal lattice points are then described by linear combinations of the reciprocal space vectors: $\mathbf{q}_{xy} = h\mathbf{a}^* + k\mathbf{b}^*$, with Miller indices (*h, k*). In the experiments, peaks occur for $Q_{xy} = |\mathbf{q}_{xy}|$. In this scheme, the three low order peaks originating from the hydrocarbon chain lattice correspond to the Miller index pairs (0,1), (1,0) and (1,1). However, a super lattice formed by the whole molecule would then have to be described by fractional indices, which are inconvenient for the present discussion. Therefore, all peaks have been indexed according to the superlattice under consideration and not according to the hydrocarbon chain lattice. The superlattice unit cell vectors, $\mathbf{a}_s$ and $\mathbf{b}_s$, are expressed as linear combinations of primitive unit cell vectors as $\mathbf{a}_s = a_1\mathbf{a} + a_2\mathbf{b}$, $\mathbf{b}_s = b_1\mathbf{a} + b_2\mathbf{b}$. Here, a_1, a_2, b_1 and b_2 are integers, making the super lattice commensurate with the hydrocarbon chain

lattice. With that, the Bragg peaks can be indexed with integer Miller indices as (h_s, k_s), corresponding to the reciprocal lattice points $\boldsymbol{q}_{xy} = h_s\, \boldsymbol{a}_s^* + k_s\, \boldsymbol{b}_s^*$. Finally, the superlattice is obtained by sampling all possible sets of (a_1, a_2, b_1, b_2) until the experimental peaks are matched. The experimental and calculated peaks peak position, Miller indices as well as the resulting supercell dimensions are summarized in the GIXD tables below.

GIXD Tables

Table S1. GIXD results of monolayers of ***lipid4*** on a ***pH 9*** subphase measured at 5 °C: Bragg peak and rod positions and their corresponding full-width at half-maximum (FWHM).

π [mN/m]	Q_{xy1} [$Å^{-1}$]	Q_{z1} [$Å^{-1}$]	Q_{xy2} [$Å^{-1}$]	Q_{z2} [$Å^{-1}$]
10, 20	1.492<br>0.024	0.0<br>0.32	1.623<br>0.023	0.0<br>0.32
30	1.492<br>0.078	0.0<br>0.32	1.623<br>0.050	0.0<br>0.32

$L_{xy1} \approx 0.9(2\pi/0.021Å^{-1}) = 269$ Å, $L_{xy2} \approx 0.9(2\pi/0.020Å^{-1}) = 283$ Å, *average crystallite size of **lipid4** at 20 mN/m ≈ 670 nm²*

$L_{xy1} \approx 0.9(2\pi/0.077Å^{-1}) = 73$ Å, $L_{xy2} \approx 0.9(2\pi/0.048Å^{-1}) = 118$ Å, *average crystallite size of **lipid4** at 30 mN/m ≈ 70 nm²*

Table S2. GIXD results of monolayers of ***Lipid4*** on a ***pH 9*** subphase measured at 5 °C: Lattice parameters a, b, c and α, β, γ, lattice distortion (d), chain tilt (t) from the surface normal, in plane area per alkyl chain (A_{xy}) and chain cross-sectional area (A_0).

π [mN/m]	a/b/c [Å]	α/β/γ [°]	distortion d	tilt t [°]	A_{xy} [$Å^2$]	A_0 [$Å^2$]
	5.018	114.10				
10, 20, 30	4.613	122.95	0.115	0.0	19.43	19.43
	4.613	122.95	NN			

Table S3. GIXD results of monolayers of ***TH4*** on ***pH 9*** *(same as **pH 10**)* subphase measured at 20 °C: Bragg peak and rod positions and their corresponding full-width at half-maximum (FWHM).

π [mN/m]	Q_{xy1} [$Å^{-1}$]	Q_{z1} [$Å^{-1}$]	Q_{xy2} [$Å^{-1}$]	Q_{z2} [$Å^{-1}$]	Q_{xy3} [$Å^{-1}$]	Q_{z3} [$Å^{-1}$]
10, 20, 30	1.264<br>0.086	0.765<br>0.33	1.371<br>0.065	0.444<br>0.33	1.561<br>0.167	0.321<br>0.35

$L_{xy1} \approx 0.9(2\pi/0.085Å^{-1}) = 67$ Å, $L_{xy2} \approx 0.9(2\pi/0.064Å^{-1}) = 88$ Å, $L_{xy3} \approx 0.9(2\pi/0.167Å^{-1}) = 34$ Å,
*average crystallite size of **TH4** at 30 mN/m ≈ 30 nm²*

Table S4. GIXD results of monolayers of ***TH4*** on ***pH 9*** *(same as **pH 10**)* subphase measured at 20 °C: Lattice parameters a, b, c and α, β, γ, lattice distortion (d), chain tilt (t) from the surface normal, in plane area per alkyl chain (A_{xy}) and chain cross-sectional area (A_0).

**π [mN/m]**	**a/b/c [Å]**	**α/β/γ [°]**	**distortion d**	**tilt t [°]**	**A_{xy} [Å^2]**	**A_0 [Å^2]**
	4.804	129.43				
10, 20, 30	5.211	123.09	0.250	31.9	23.88	20.27
	5.933	107.47				

headgroup peak at 1.321 Å^{-1} , *d* = 4.756 Å

Table S5. GIXD results of monolayers of ***TH14*** on ***pH 9*** subphase spread at 30 °C, measured at 20 °C: Bragg peak and rod positions and their corresponding full-width at half-maximum (FWHM).

**π [mN/m]**	**Q_{xy1} [Å^{-1}]**	**Q_{z1} [Å^{-1}]**	**Q_{xy2} [Å^{-1}]**	**Q_{z2} [Å^{-1}]**	**Q_{xy3} [Å^{-1}]**	**Q_{z3} [Å^{-1}]**
10	1.036	1.152	1.269	0.973	1.464	0.179
	0.082	0.34	0.017	0.34	0.026	0.34
20	1.039	1.140	1.273	0.834	1.471	0.306
	0.081	0.34	0.025	0.34	0.172	0.34
30	1.045	1.112	1.275	0.847	1.467	0.265
	0.085	0.34	0.028	0.34	0.072	0.34

$L_{xy1} \approx 0.9(2\pi/0.084Å^{-1}) = 67$ Å, $L_{xy2} \approx 0.9(2\pi/0.025Å^{-1}) = 226$ Å, $L_{xy3} \approx 0.9(2\pi/0.071Å^{-1}) = 80$ Å,
*average crystallite size of **TH14** at 30 mN/m = 130 nm^2*

Table S6. GIXD results of monolayers of ***TH14*** on ***pH 9*** subphase spread at 30 °C, measured at 20 °C: Lattice parameters a, b, c and α, β, γ, lattice distortion (d), chain tilt (t) from the surface normal, in plane area per alkyl chain (A_{xy}) and chain cross-sectional area (A_0).

**π [mN/m]**	**a/b/c [Å]**	**α/β/γ [°]**	**distortion d**	**tilt t [°]**	**A_{xy} [Å^2]**	**A_0 [Å^2]**
	5.059	136.2				
10	6.197	121.9	0.383	51.3	30.68	19.25
	7.149	101.9				
	5.040	136.2				
20	6.175	122.1	0.386	49.8	30.48	19.67
	7.136	101.7				
	5.043	135.9				
30	6.152	121.9	0.377	49.1	30.32	19.85
	7.079	102.2				

Table S7. GIXD results of monolayers of ***TH12*** on ***pH 10*** subphase measured at 20 °C: Bragg peak and rod positions and their corresponding full-width at half-maximum (FWHM).

π [mN/m]	***Q_{xy1} [Å^{-1}]***	***Q_{z1} [Å^{-1}]***	***Q_{xy2} [Å^{-1}]***	***Q_{z2} [Å^{-1}]***	***Q_{xy3} [Å^{-1}]***	***Q_{z3} [Å^{-1}]***
15, 25, 35	1.013<br>0.024	1.105<br>0.33	1.275<br>0.017	0.905<br>0.34	1.465<br>0.016	0.200<br>0.34

$L_{xy1} \approx 0.9(2\pi/0.021Å^{-1}) = 269$ Å, $L_{xy2} \approx 0.9(2\pi/0.012Å^{-1}) = 471$ Å, $L_{xy3} \approx 0.9(2\pi/0.011Å^{-1}) = 514$ Å,
*average crystallite size of **TH12** at 30 mN/m ≈ 1200 nm^2*

Table S8. GIXD results of monolayers of ***TH12*** on ***pH 10*** subphase measured at 20 °C: Lattice parameters a, b, c and α, β, γ, lattice distortion (d), chain tilt (t) from the surface normal, in plane area per alkyl chain (A_{xy}) and chain cross-sectional area (A_0).

**π [mN/m]**	**a/b/c [Å]**	**α/β/γ [°]**	**distortion d**	**tilt t [°]**	**A_{xy} [Å^2]**	**A_0 [Å^2]**
	5.025	137.30				
15, 25, 35	6.324	121.41	0.404	50.2	31.17	19.93
	7.267	101.29				

Table S9. GIXD results of monolayers of ***DiTT4*** on ***pH 10*** subphase measured at 20 °C: Bragg peak and rod positions and their corresponding full-width at half-maximum (FWHM).

π [mN/m]	***Q_{xy1} [Å^{-1}]***	***Q_{z1} [Å^{-1}]***	***Q_{xy2} [Å^{-1}]***	***Q_{z2} [Å^{-1}]***	***Q_{xy3} [Å^{-1}]***	***Q_{z3} [Å^{-1}]***
20	1.397<br>0.045	0.547<br>0.34	1.457<br>0.018	0.0<br>0.34		
30, 40	1.380<br>0.014	0.119<br>0.34	1485<br>0.016	0.517<br>0.29	1.528<br>0.019	0.398<br>0.34

$L_{xy1} \approx 0.9(2\pi/0.007Å^{-1}) = 808$ Å, $L_{xy2} \approx 0.9(2\pi/0.011Å^{-1}) = 514$ Å, $L_{xy3} \approx 0.9(2\pi/0.015Å^{-1}) = 377$ Å,
*average crystallite size of **DiTT4** at 30 mN/m ≈ 2600 nm^2*

Table S10. GIXD results of monolayers of ***DiTT4*** on ***pH 10*** subphase measured at 20 °C: Lattice parameters a, b, c and α, β, γ, lattice distortion (d), chain tilt (t) from the surface normal, in plane area per alkyl chain (A_{xy}) and chain cross-sectional area (A_0).

π [mN/m]	***a/b/c [Å]***	***α/β/γ [°]***	***distortion d***	***tilt t [°]***	***A_{xy} [Å^2]***	***A_0 [Å^2]***
	5.271	117.13				
20	5.054	121.43	0.056	24.6	22.73	20.66
	5.054	121.43	NN	NN		
	4.694	125.51				
30, 40	5.051	118.83	0.118	19.6	21.37	20.13
	5.197	115.66				

Table S11. Experimental and calculated GIXD peak positions for ***DiTT4*** on ***pH 10*** subphase measured at 20 °C. See main text for the description of the graphical illustration of the molecular superlattice.

Q_{xy} ^{Experimental} [Å^{-1}]	Q_{xy} ^{Calculated} [Å^{-1}]	Miller Indices (h_S, k_S)	b/c^{Supperlattice} [Å]	α^{Supperlattice} [°]	A^{Supperlattice} [Å^2]
0.825	0.827	(-1, 2); (1, -2)	10.102	125.5	128.22
0.992	0.990	(0, -2); (0, 2)	15.592		
1.127	1.126	(1, 1); (-1, -1)			
1.213	1.213	(1, -3); (-1, 3)			
1.247	1.248	(2, -2); (-2, 2)			
1.304	1.304	(-2, 1); (2, -1)			
1.380	1.379	(-2, 3); (2, -3)			
1.485	1.485	(0, -3); (0, 3)			
1.528	1.528	(2, 0); (-2, 0)			
1.563	1.563	(1, 2); (-1, -2)			
1.657	1.657	(1, -4); (-1, 4)			

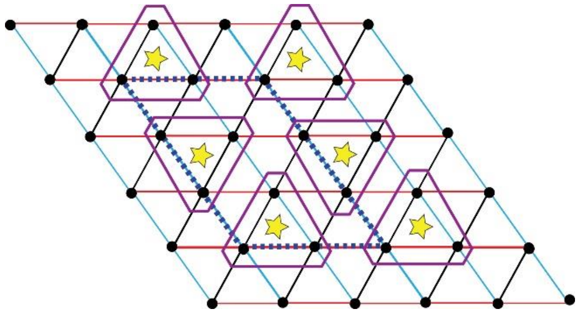

References:

1. Wölk, C.; Drescher, S.; Meister, A.; Blume, A.; Langner, A.; Dobner, B., General Synthesis and Physicochemical Characterisation of a Series of Peptide-Mimic Lysine-Based Amino-Functionalised Lipids. *Chem. Eur. J.* **2013,** 19, 12824 – 12838.
2. Wölk, C.; Heinze, M.; Kreideweiß, P.; Dittrich, M.; Brezesinski, G.; Langner, A.; Dobner, B., Synthesis and DNA transfection properties of new head group modified malonic acid diamides. *International Journal of Pharmaceutics* **2011,** 409, 46–56.
3. Wölk, C.; Janich, C.; Pawlowska, D.; Drescher, S.; Meister, A.; Hause, G.; Dobner, B.; Langner, A.; Brezesinski, G., Lamellar versus Micellar Structures— Aggregation Behavior of a Three-Chain Cationic Lipid Designed for Nonviral Polynucleotide Transfer. *ChemPhysChem* **2015,** 16, 2115-2126.
4. Andreeva, T. D.; Petrov, J. G.; Brezesinski, G.; Moehwald, H., Structure of the Langmuir monolayers with fluorinated ethyl amide and ethyl ester polar heads creating dipole potentials of opposite sign. *Langmuir* **2008,** 24, 8001–8007.
5. Flach, C. R.; Gericke, A.; Mendelsohn, R., Quantitative Determination of Molecular Chain Tilt Angles in Monolayer Films at the Air/Water Interface: Infrared Reflection/Absorption Spectroscopy of Behenic Acid Methyl Ester. *J. Phys. Chem. B* **1997,** 101, 58.
6. Mendelsohn, R.; Brauner, J. W.; Gericke, A., External infrared reflection absorption spectrometry of monolayer films at the air-water interface. *Annu. Rev. Phys. Chem.* **1995,** 46, 305.
7. Als-Nielsen, J.; Jacquemain, D.; Kjaer, K.; Leveiller, F.; Lahav, M.; Leiserowitz, L., Principles and applications of grazing incidence x-ray and neutron scattering from ordered molecular monolayers at the air-water interface. *Physics Reports* **1994,** 246, (5), 251-313.
8. Stefaniu, C.; Vilotijevic, I.; Santer, M.; Varon Silva, D.; Brezesinski, G.; Seeberger, P. H., Subgel Phase Structure in Monolayers of Glycosylphosphatidylinositol Glycolipids. *Angew. Chem. Int. Ed.* **2012,** 51, 12874 – 12878.
9. Kittel, C., *Introduction to Solid State Physics* 6th ed.; Wiley: New York, 1986.
